# Health Impact Assessment Practice and Potential for Integration within Environmental Impact and Strategic Environmental Assessments in Italy

**DOI:** 10.3390/ijerph111212683

**Published:** 2014-12-08

**Authors:** Nunzia Linzalone, Giorgio Assennato, Adele Ballarini, Ennio Cadum, Mario Cirillo, Liliana Cori, Francesca De Maio, Loredana Musmeci, Marinella Natali, Sabrina Rieti, Maria Eleonora Soggiu, Fabrizio Bianchi

**Affiliations:** 1 Institute of Clinical Physiology, National Council of Research, via Moruzzi 1, 56127 Pisa, Italy; E-Mails: liliana.cori@ifc.cnr.it (L.C.); fabrizio.bianchi@ifc.cnr.it (F.B.); 2 Regional Agency for the Protection of the Environment, Apulia Corso Trieste 27, 70126 Bari, Italy; E-Mail: g.assennato@arpa.puglia.it; 3 Regional Public Health Service, Emilia-Romagna Viale Moro 21, 40127 Bologna, Italy; E-Mails: ABallarini@Regione.Emilia-Romagna.it (A.B.); MNatali@regione.emilia-romagna.it (M.N.); 4 Regional Agency for the Protection of the Environment, Piedmont via Sabaudia 164, 10095 Grugliasco, Italy; E-Mail: ennio.cadum@arpa.piemonte.it; 5 Institute for Environmental Protection and Research, via Vitaliano Brancati 48, 00144 Roma, Italy; E-Mails: mario.cirillo@isprambiente.it (M.C.); francesca.demaio@isprambiente.it (F.D.M.); sabrina.rieti@isprambiente.it (S.R.); 6 Istituto Superiore di Sanità, Viale Regina Elena 299, 00161 Roma, Italy; E-Mails: loredana.musmeci@iss.it (L.M.); mariaeleonora.soggiu@iss.it (M.E.S.)

**Keywords:** public health policy, Health Impact Assessment, Environmental Impact Assessment, Strategic Impact Assessment, impact assessment tools, policy decision-making

## Abstract

Avoiding or minimizing potential environmental impact is the driving idea behind protecting a population’s health via Environmental Impact Assessments (EIAs) and Strategic Environmental Assessments (SEAs). However, both are often carried out without any systematic approach. This paper describes the findings of a review of HIA, EIA and SEA experiences carried out by the authors, who act as institutional competent subjects at the national and regional levels in Italy. The analysis of how health is tackled in EIA and SEA procedures could support the definition of a protocol for the integration of HIA with EIA and SEA. Although EIA and SEA approaches include the aim of protecting health, significant technical and methodological gaps are present when assessing health systematically, and their basic principles regarding assessment are unsatisfactory for promoting and addressing healthcare concepts stated by the WHO. HIA is still poorly integrated into the decision-making process, screening and monitoring phases are only occasionally implemented, and operational details are not well-defined. The collaborative approach of institutions involved in environment and health is a core element in a systematic advancement toward supporting effective decisions and effective protection of the environment and health. At the Italian national level, the definition of guidelines and tools for HIA, also in relation with EIA and SEA, is of great interest.

## 1. Introduction

There has been substantial interest worldwide in promoting the inclusion of healthcare within policy in order to achieve Health in All Policies (HiAP) goals, since its first acknowledgment in Europe in 2006 [1]. A Health Impact Assessment (HIA) [2] is a means for realizing the principles of HiAP. HiAP and HIA have similar aims and they share the recognition of the need for all sectors to work together to advance the social and health development of populations [3]. HIA is recognized as an appropriate tool for including health concerns in the evaluation of various proposals across levels of application and sectors [4,5,6]. Since the early evolution of HIA, the practice was developed to provide a flexible tool adaptable to different contexts, sectors, and resource availability [7], while lack of knowledge is a limiting factor in HIA implementation [8,9]. This is why capacity-building in HIA has been addressed by different levels of government organizations in the public health sector, environmental sector, and other sectors involved in the process [10,11,12,13]. While no restrictions for the HIA application exist in any sector [14], an HIA does not always have the potential to be useful nor it is required when the potential health impact is obvious and known [9]. Therefore, screening is used to systematically decide if and when an HIA is required. More recently, the focus of HIA on equity and public participation has been explored more systematically [10]. Depending on a wide range of applications, the production of technical and methodological HIA guidance and specific tools has grown [15]. Lessons learned from HIA applications have been usefully collected under numerous HIA web resources [16]. Different criteria can be used to classify existing case studies and a review of national experiences is often a suitable way to define a general standard at the country level [3,17,18,19]. The HIA approach, used as a stand-alone impact assessment tool or integrated into procedures for assessing the environmental impact of projects and plans in the environmental setting, *i.e.*, the Environmental Impact Assessment (EIA) or Strategic Environmental Assessment (SEA), have the potential to incorporate health considerations both in public health and in environmental health decision-making processes [20,21,22]. 

EIA and SEA both envisage the assessment of health impacts. Nevertheless, it is important to note that in the past, in Italy, the absence of specific protocols for HIA in EIA and SEA have resulted in a lack of involvement of public health institutions with statutory responsibilities in the evaluation procedures, mainly the National Health Institute (ISS) and the Institute for Environmental Protection and Research (ISPRA). The ISS is the Italian technical-scientific body of the Ministry of Health responsible for public health, and the Department of Environment and Health of ISS conducts studies on the relationship between environmental contamination and its effects on health. Since the first application of the EIA directive at the end of the 1980’s, in collaboration with WHO [23,24] the ISS has tackled the problem of integrating the health component within the EIA and has organized seminars and courses for the staff of the national health system and Regional Environmental Agencies (ARPA). Some critical elements have been identified. In particular, application of the EIA directive has highlighted the complexity of the assessments in reference to the multidisciplinary nature of the experts needed to address the issue of a predictive approach to preventing adverse health and environmental effects. Very often, the health component in the EIA has been considered only as a description of the health profile of the local population involved, and no prospective assessment has been produced in accordance with the procedures of risk assessment. The lack of specific guidelines as well as the scarce involvement of health-scientific institutions within the evaluation procedures have been major limits for the proper consideration of impact on health. In recent years, greater collaboration between health and environmental institutions, with various commitments by the Ministry of Health, permitted development of methods and procedures for a more comprehensive approach. The case studies and experiences presented in this paper, selected for their national relevance, demonstrate the results of inter-institutional collaborations. The paper analyzes cases of HIA or health assessment provided within the EIA and SEA in Italy in order to identify common key points in health impact assessments of policies, plans or projects. The aim is to outline gaps and needs for the definition of a standard model to compare the three approaches and achieve better inclusion of health considerations in strategic planning and project evaluation.

## 2. Methods, Selection of Cases and Framework for HIA Case Evaluation

The experience of impact assessments presented here (both case studies done by the authors or reviews of EIA and SEA reports) were chosen because they are representative of the issues relating to the activities of health impact assessment and their effective integration with the components of environmental assessment. Three case studies of HIA were analyzed [25,26,27]; all the EIA and SEA national procedures of new projects, plans or programs were screened for the presence/absence of a health chapter within the environmental report; two EIAs concerning strategic national projects were included as relevant case studies of integration of HIA in EIA. The national mandatory EIA database was analyzed starting from 1989, when EIA was implemented in a systematic way in Italy, and the SEA database starting from 2008, when ISPRA started to support the Ministry of the Environment in the evaluation of national SEA procedures.

An analytical framework describing general HIA practice, coded by the Gothenburg Consensus Paper, was used to analyze HIA applications. The framework was outlined by a theoretical search within the MonITER Project [28], which compared existing HIA frameworks developed by other authors [20,29] and covers the seven following aspects: (a) model of health; (b) focus of HIA; (c) application level; (d) integration with other assessment forms; (e) participation; (f) type of evidence (g) added value to the proposal. To facilitate the review, a set of questions shown in Table 1 was used. The questions addressed have been discussed in the HIA literature by other authors [19]. The findings of the review are shown in Table 2, and key issues are discussed in Section 4.

**Table 1:** Questions for analyzing case studies regarding the key aspect of health impact assessment.

1	**Conceptual understanding of health** What definition of health was considered in appraisal? Has the definition of health adopted been explicated and agreed on/discussed by the steering committee? Is the definition of health outlined within the circulated documents? Where is it described in the process development?
2	**Main concern addressed** What is the object of assessment? Are unknown impacts considered by the HIA? Identification of indirect impacts, focusing the determinants of health? Consideration of differently affected subgroups from an equity and social justice point of view? Prediction of direct impact on physical health stemming from defined environmental risks?
3	**Application level** When is the assessment made in relation to the decisional process? Is the area smaller/larger than a municipality? Is the assessment high/medium/low resource-consuming? Is the proponent from a government institution? Local/regional/national?
4	**Integration of IA types** Does the assessment cover more than one different kind of impact (*i.e.*, health and environment)? Are cross-cutting themes related to impacts combined in one process (*i.e.*, sustainability evaluation)? Are equity or social justice assessed? Are relations with policies/plans affecting widespread health and well-being determinants reviewed/mentioned/assessed?
5	**Involvement of stakeholders** Is there any kind of participation? When? Who was involved? Local government/authorities, advisory authorities, local communities, subgroups of society?
6	**Type of evidence** Qualitative data? A mix of qualitative and quantitative? What kind of health data are used: literature, toxicology-based, epidemiology-based, on routine or study sample data, interviews, questionnaire? Information provided: magnitude of impact, baseline health status, risk measures?
7	**Added value of HIA ** What is the assessment carried out for? Institutional decision-making support (evaluating interventions, prospective assessment of projects), advocacy by subgroups (influencing decision and implementation, understand and contribute to decision-making)? Is the process voluntary or required by regulation (undertaken by the proponent, decision-maker, external organization, community-led)? Is the decisional component included in the process? Are conflicting parties included? Is there a monitoring phase? On the implementation of the recommendations? On the reduction of impact? Other aims?

## 3. Results and Discussion

### 3.1. State of the Art on Health Impact Assessment

IFC-CNR undertakes numerous epidemiological studies in polluted areas in Italy [30]. The HIA approach has been applied by IFC-CNR in case studies that encompass levels of assessment from desktop [26] to comprehensive [25,27]. Also, specific methodological issues of HIA practice have been covered to support the definition of a general best practice [28,31]. Specific research action was addressed to define the relevance of HIA at the local level, integrating the view and knowledge of public officials in the Regional administration [32].

**Table 2:** Analytical framework: key aspects and questions addressed in HIA case studies.

Key Dimensions	Study Reference		
	Bianchi *et al.*, 2006 [25]	Adam *et al.*, 2014 [26]	Linzalone *et al.*, 2014 [27]
1. Mode l of health	Biomedical model ▪ Mortality and morbidity rates in the exposed population	Biomedical model and exploration of socioeconomic determinants of health ▪ Expected risks in the exposed population ▪ Consideration of wider determinants modified by upstream risk factors	Biomedical model and exploration of socioeconomic determinants of health ▪ Mortality and morbidity rates in the exposed population ▪ Expected risks in the exposed population ▪ Consideration of socio-economic determinants
2. Focus of HIA	▪ Prospective assessment of physical impacts from different plan options ▪ Equity is not specifically addressed, the consideration of the distribution of effects is included.	▪ Prioritization of unknown determinants and related interacting policy to evaluate the effects of interventions ▪ Equity is not specifically addressed, access to healthcare is included.	▪ Prospective assessment of physical impacts from different plan options ▪ Equity is not specifically addressed, the consideration of the distribution of effects is included.
3. Application level	▪ The project of building a new plant was judged about the potential health impacts on the local population. ▪ Provincial level area was scoped. The assessment was high resource intensive.	▪ The wide impact of a current regional policy was scoped. A sample of a vulnerable group was scoped. The assessment was medium low resource intensive.	▪ The decision about empowering the existing was informed. Municipal level area was scoped. The assessment was medium resource intensive.
4. Integration	▪ A mandatory EIA was coupled with the assessment of the health impact on exposed communities.	▪ None	▪ A voluntary assessment was carried out on health, environment and socioeconomic impacts.
5. Participation	▪ The decisional component was included only in the negotiation phase, when the assessment had been concluded.	▪ No local dissemination of findings to decision makers was planned and the specific research results were not opportunely used within the regional policy framework.	▪ Large participation was planned. Decision-makers, stakeholders and communities were involved with different methodologies, within each HIA stage.
6. Type of evidence	▪ Environmental and health information were integrated to define the best alternative location of the plant. ▪ The baseline health status was provided for the communities. ▪ Residential exposure was modeled based on the past point source emission scenario. Literature and routine health data were used to scope the impacts.	▪ Literature health data were used to scope the impacts. Expert opinion was used to prioritize the impacts by questionnaire interview.	▪ Environmental and health information were integrated to assess doubling the plant’s power or not. ▪ The health risk of potential outcomes was estimated for the communities ▪ Individual exposures were modeled based on the past point source emission scenario. Literature and routine health data were used to scope the impacts. Focus groups helped identify additional concerns about specific determinants of health.
7. Added value to the proposal	▪ The municipality joined the citizens’ action to advocate for a fair decision regarding the local waste management plan. ▪ The decision-making process halted because stakeholders and communities complained about not being involved earlier.	▪ Research transfer to those involved was the outcome of a voluntary study on the impact of ionizing radiation use in clinical practice.	▪ A funded research project supported the decision to be made on the local waste plan. ▪ The decision was informed with the HIA results and a positive conclusion was drawn rapidly. ▪ Monitoring recommendations was not carried out but indicators were developed in one case.

Since 2008 there has been an ongoing special collaboration with regions and municipalities implementing the requirements of UN Agenda 21 [33] to raise awareness in municipalities and citizens regarding HIA foundations. Findings from HIA case studies [25,26,27] are shown in Table 2.

The development of HIA stages looks patchy, with major strengths in the characterization of population profiles [25,27], the policy context [26,27], the stakeholders’ involvement, political commitment in the assessment process [26], the data needed, and quantification of impact [25,26]. A qualitative assessment of impact was also experimented to link the determinants of health to the final outcome through risk factors, using the causal chain approach. A workshop session was carried out as a means of stakeholder consultation to prioritize determinants and sectors for programming focused interventions [26].

The cases analyzed show how HIA has been handled and emerging issues are discussed. The screening of the proposal and the monitoring of outcomes appear formal to some extent. While the screening process usually determines whether it would be appropriate to conduct an HIA of the chosen policies, in the two cases of assessing the impact of a working waste incinerating plant [26] and of the proposed project of a new incinerator [25], it has been finalized to collect available data and provide a report with the likely impacts. Furthermore, an attempt has been made to support the decisional component to judge the HIA opportunity, developing a prototypal screening-scoping tool [28] subsequently applied within a real setting [31]. The piloting phase showed that the tool was weak in defining a cut-off to “reject” HIA, so its effectiveness was limited. However, its application in several contexts showed that it was always very helpful in the preliminary definition of the main impacts and the identification of cross-issues related to the proposal. Further work is needed to fit the tool for use within specific sectors. Regarding the monitoring phase, it has never been implemented. In one case [27], indicators of impacts have been developed for future follow-up and monitoring, including a minimum set of recommendations about stakeholder roles. However, a monitoring plan for the implementation was not provided.

### 3.2. Health Assessment in EIA Reports 

Among the 10,720 requirements accounted for in the period 1989–2013, 122 refer to “public health” and of these only nine were directly related to health, while the others dealt with air, water, noise, electromagnetic fields, *etc.* The requirements directly related to health were: four epidemiological studies, three analyses of mortality and morbidity, and two risk assessments. 

From 2008 to 2013, 131 EIAs have been analyzed by ISPRA, and for 91 of these proposals ISPRA was also required to check the “health component”. Health is not systematically present in the analyzed “Environmental Impact Study” (EIS, *i.e.*, the environmental report about the proposal). For most project categories, particularly those with a greater number of projects (road and railway infrastructures, power lines and pipelines), the health component is often not present. For example, for road infrastructure projects EIS includes a health chapter in only 23% of the analyzed proposals. EIS for other project categories, with fewer projects but still hazardous for health (refinery plants, hazardous waste treatment plants, energy power plants), are provided with a specific chapter for health. In general, explicit health assessment is absent in 32% of the analyzed proposals. Often the context is not adequately described, and both the pollutants and the population are poorly characterized. Exposure quantification, forecast of impacts and food chain contamination are seldom analyzed. A health monitoring plan is never present.

### 3.3. Health Issues in SEA Reports

From 2008 to 2013, ISPRA supported the Ministry of the Environment in the evaluation of 18 national and 30 regional SEA procedures. The objects of the assessment ranged from national electric networks, to water management and land planning. Regional SEA include plans/programs on waste, air quality, transport, telecoms, fishing, and mining. The health component is addressed as a goal of sustainability and health is part of a general context exploration. Health assessment does not refer to a standard process in terms of positioning within the assessment procedure (either in the scoping or assessment phase). The main shortcomings found in SEA (National and Regional), are mainly in line with recent findings [34]:

- Absence/lack of characterization of the environmental factors that directly and/or indirectly affect the health of exposed people.
- Absence/lack of identification and characterization of the potential risks associated with the actions contained in the plan/program.
- Absence/poor evaluation of the effects on health deriving from the implementation of plan/program.
- Lack of consideration for prevention and reduction of effects on health.

### 3.4. Piedmont Region Case Study. Environmental Impact Assessment of the New High-Speed Railway Turin-Lyon

The new high-speed railway between Turin (in Italy) and Lyon (in France) is one of the largest national projects foreseen by Italian Government in the next few decades. The EIA included, among other compulsory requirements, the obligation for the proponent Company (Lyon-Turin Ferroviaire, LTF), to “Analyze the aspects related to public health according to the reliable methods of Health Impact Assessment”. Due to the absence of a national HIA standard, the methodology was provided to the Company by experienced regional officials. It included (a) preliminary analysis of the health status of the affected population; (b) a comprehensive 5-step HIA based on regionally published guidelines [35]. The envisaged HIA includes both a quantitative and a qualitative assessment of impact; in addition, the different tasks for the involvement of the local Environmental and Health Agencies are explored. Critical aspects of this process can be summarized as follows:

1. The final project approval was decided by an EIA procedure and HIA was required as enclosed requirement, to monitor impact during and after their occurrence, over a long period of time.
2. The preventive knowledge of the health status of the population did not inform the HIA screening phase (the decision about the feasibility of the Project was made a priori and independently from the evaluation of the health status of the population at baseline).

This is the first HIA procedure required by law in Italy, formally published in the Official Gazette of the Italian republic [36] (quoting CIPE requirement N.130: “[…] investigate aspects related to public health according to the accredited HIA models”). However the absence of a national standard for HIA may influence the achievable results with this specific tool, and the goal of introducing a positive modification to the health impacts of the proposal may be jeopardized. The lack of an “open” steering committee performing the HIA (carried out by the Company) and judging on selected methods for the assessment, times and procedures, are serious obstacles to the credibility of the HIA results and positive conclusion.

### 3.5. Apulia Region Case-Study. Gaps in Preventing Environmental-Health Risk from ILVA Plant

For more than 40 years the ILVA steelworks has contributed significantly to the increased environmental levels of pollutants. The lack of coverage of health issues within the Integrated Prevention Pollution and Control (IPPC) permit process (entirely based on the negotiation of selected Best Available Techniques for each shop of the plant) resulted in the intervention of the Local Prosecutor. A team of expert epidemiologists studied several short-term and long-term effects of the ILVA emissions on the general population. As a consequence, a regional law was approved requiring the local environmental and health institutions to perform a so-called “Assessment of Health Damage” (VDS) and the assessment of health impact before and after the adoption of new requirements (year 2010 *vs.* year 2016) was carried out. The cancer and non-cancer risks for inhalation were estimated for the exposed population and showed a decrease compared to the baseline scenario. In order to provide a comparison with a standard HIA procedure a stepwise process can be summarized as follows:

1. Pre-screening for the definition of a base of evidence needs and the role of the local environmental and health institutions.
2. Screening to provide the systematic analysis of current evidence on the extent of environmental pollution as well as of local health descriptors.
3. The scoping and assessment phases were led by the ARPA of the Apulia Region, the regional Health Agency and the Taranto health local unit. The VDS consisted in the independent parallel evaluation of industry-related health outcomes (mortality, cancer registry, hospital discharge data) and risk assessment based on the EPA residual risk approach [37].
4. A monitoring activity is due yearly according to the approved regulations (Integrated Environmental Authorization), which include a communication report.

For the first time, the combined use of health-based approaches (both epidemiological and risk-assessment) was used in the context of IPPC. However, a communication plan is lacking, although public audiences were held to inform the local stakeholders. The highly controversial case of ILVA supports the adoption of a transparent scoping phase to define the participation of the relevant parties, the resource available for the comprehensive assessment and prioritization of the health indicators to be monitored over time. The inclusion of the HIA within the permit process and not after, as in the ILVA case, would be appropriate for prevention purposes.

## 4. Discussion and Analysis

### 4.1. Definition of a Model of Health and Outcome Assessment

HIA principles stem from, and are guided by, the broad societal values of democracy, sustainability and equity [3]. HIA definition is based on a broad model of health, which proposes that economic, political, social, psychological, and environmental factors determine population health. In Italy the earliest HIA was based on an a priori biomedical model of illness and disease, and consequently the investigated outcomes were those quantified by mortality and morbidity rates or through risk estimates for selected outcomes. The evolution of the HIA applications in Italy have led to inclusion of a socio-economic “holistic” model of health, contributing to clarification of the importance of risk factors acting on the wide determinants of health, in different non-health sectors, in a full-chain model of impact [38].In the specific context of the mandatory evaluation of projects, plans and programs via EIA and SEA procedures, an explicit definition of what the sustainability means for the assessment of human health should be included among the terms of reference in the health report/chapter. In particular, a clear definition of the concept of health allows identification of the amount of resources in terms of time and necessary data, quality of health information on the population, skills and expertise necessary for a full assessment of health. 

### 4.2. Focus of Assessment and Definition of Methods

Different traditions of environmental and public health developed three different trends in HIA. One typical public health perspective on the environment is a “pollution-driven” concept of environmental effects on human health [39]. Another public health policy trend broadens the term “health”, taking a social view of health focusing on wider determinants. The third trend, HIA focused on health equity, was proposed to enhance the equitable distribution of effects of proposals (particularly policy proposals) on the health of populations [40]. In Italy, when the HIA is carried out as an independent procedure, a distinction regarding the main focus has been highlighted as to whether the proposal is a policy, plan or project.

The assessment of a policy to protect health has been considered more suited to the analysis of the broad determinants of health, and a causal chain can be drafted to identify all possible transversal factors affecting final outcomes. In this sense, the final recommendations take into account distal risk factors that modify health in an indirect way. An equity issue is explicitly introduced as a factor that can modify the final risk and can be addressed at different points in the causal chain [26].

When a plan is assessed for potential impact, a social view of health facilitates identifying its effects on culture, knowledge, integration, participation, trust and local perception. Recommendations from HIA should include the identification of participants to be involved and interventions to address area-specific impact [27]. 

In the project assessment of a new plant building [25], clear identification of the exposed population and the risk for relevant health outcomes were based on community profiling. Moreover, a micro-geographic study on the exposed population supported a scenario analysis oriented towards the selection of an alternative collocation of the plant, minimizing the negative effects. In this case, an equity focus is implicitly considered because the impact on the highly affected population or the vulnerable groups in the study area is scoped.

Human health is explicitly considered to be among EIA and SEA fundamentals. In the assessment phase, requirements of compliance can be stricter than the regulations demand, when a possible risk is forecast for the environment and the population. Nonetheless, currently the health component is disregarded, while focusing on the natural and physical environment. In fact, the approach to health protection is that minimizing the concentrations of pollutant (*i.e.*, complying with the regulatory limits) has a protective effect on health. However, an analysis of the effects from multiple and aggregate exposures on the health of a given population is not always provided. Currently, this approach drives almost all the procedures carried out within the mandatory assessment of proposals. In this situation, technical regulations and guidelines have the potential for improving knowledge about impacts. In particular, clarification is needed regarding the theoretical definition of health and describing data needs, available methodologies for quantification of impacts and special threats requiring a more thorough assessment. However, the definition of a minimum standard is challenging in Italy, due to regional heterogeneity of the availability of environmental data.

### 4.3. Positioning of HIA and Application Level Are Key Issues in Selecting Stakeholders

HIA is typically introduced within the policy and planning cycle after a draft proposal has been developed, but before that proposal is implemented. Openness during the scoping phase will determine the inclusion of different stakeholders and better identification of areas of interest and affected populations [41]. 

In Italy, HIA is poorly implemented prospectively. Among the barriers identified is the incomplete knowledge about HIA’s potential on the part of decision-makers and by public administrators as well as a piecemeal decision-making process [42].

When implemented in a project proposal, the screening phase has provided the definition of the relevance of impact and the lists of stakeholders to be included in the process [25]. This approach has limited the contribution of HIA to defining alternatives to the proposal’s implementation, and the decision was made previously. However, the HIA was relevant to introducing changes in the project proposal, minimizing the negative impact.

In the definition of a new plan [27], HIA was undertaken with an open approach, showing its potential to drive the scoping phase. In consideration of the broad range of interests involved, local stakeholders were invited to participate in meeting sessions with the leading HIA group for the definition of boundaries and extension of the assessment. However, it should be considered that creating collaboration among stakeholders required time and resources. The political context was critical in planning the HIA. The political and administrative component needed a special engagement process, often requiring the adaptation of time scheduled to carry out the process. In conclusion, a planned decision was re-analyzed as the HIA process included the decisional component from the beginning. The realization of the collaboration was fundamental for the accessibility of the area-specific data. 

The analysis of a policy, when addressed at the regional level, creates an opportunity for enhancing the effect of reduction of risk factors, acting on a large population affected by the policy [26]. The definition of the direct connections of the policy with the determinants of health offers different entry points to act on the final health outcome. In particular, non-health-related sectors that indirectly contribute to health and wellbeing can share responsibility in promoting health. 

EIA and SEA require the proponent of a project to provide a description of the context, including social, economic and cultural details of the proposal. When a system for recording these data is in place and is managed by the local public administration, a comprehensive assessment of a proposal is feasible in due time. Thus the positioning of the assessment process after a draft proposal is provided can clarify the nature of the potential impact on the health of the exposed population before a project is accepted. 

### 4.4. HIA Integration in EIA and SEA

The issue of integration of health assessment is usually used in the discussion of whether it is desirable or necessary to add the HIA tool to those of EIA and SEA [43]. Independent HIA has been criticized in the past both for overlapping of similar activities and for requiring additional resources. On the other hand, integration of impact assessment tools is common in sectors such as economy, insurance, transport, urban design, and employment [44,45]. 

When a prospective HIA was explicitly required by an institutional subject or when it was carried out within a research framework, the integration of information for a comprehensive assessment of impact was possible [25,27]. In both cases the HIA leading group provided epidemiological competencies and an HIA background. Different point and linear sources within the study area were analyzed to identify the contribution for the overall population [25] and individual risk [27]. The entire process covered a period lasting longer than 1 year. 

In EIA and SEA, what is challenging for the completion of the health assessment by the proponent is the definition of proposal alternatives when the context data are not available. This is the case for a large number of EIAs and SEAs in Italy. To fulfill the mandate of assessing the impact on human health, a great deal of non-specific data are always provided regarding pollutants, expected effects, *etc.* Efforts have been made by the Ministry of Environment and ISPRA to gather national resources of environmental data [46] and to define a checklist of the minimum information required to draft the Environmental Impact Statement (EIS) [47]. In some regional contexts, health surveillance and environmental monitoring data have a good level of accessibility and quality. In the Lombardy Region, easy data access has facilitated the adoption of a methodological framework to carry out HIA in EIA [48].

### 4.5. Stakeholder Involvement, Timing of Participation and Opportunity to Add Value

According to the Italian laws on EIA and SEA, and coherently with European Directives, the competent authority will examine the comments received on the draft proposal, after a period established for stakeholder consultation. In HIA methodology the participation and involvement of stakeholders are included in most of the phases, with special attention to screening, scoping and the production of recommendations. When a participatory scoping phase was performed to examine different proposals, a committee, including local participants, was established with the task of evaluating the relevance of impacts by using a checklist [31]. Another project developing an HIA of a new local waste plan provided a systematic framework for the engagement of the community through assemblies and thematic working groups [27]. Those two frameworks show critical aspects of participation; in the first case, participation is limited to a consultation of a number of “key informants”; in the second, stakeholder participation is possible and required. HIA methodology, as a tool for reinforcing the evaluation of health components of EIA and SEA, could provide the competence to properly place the evaluation in a social context, involving stakeholders and understanding their attitude towards the proposed project or plan. This could have substantial added value for the assessment. 

## 5. Conclusions 

The fundamental elements of HIA practice have been analyzed to identify the characteristics of health assessment performed in Italy, in voluntary and mandatory procedures. The reviewed cases of HIA, EIA and SEA health reports and relevant national EIA cases showed that health is commonly stated according to the WHO definition, recognizing the potential to protect and promote human health. Differences are introduced in the focus of the health assessment process, mainly according to the distinction between voluntary and mandatory assessment of impacts. A different level of application (*i.e.*, policy, plan or project) determines a different selection of methods, use of evidence, collaborations, and elements that characterize the focus of the assessment. Adopting a broader involvement of stakeholders always provides more opportunities for data collection enabling area-specific quantification of impacts.

However, integrating health assessment within a mandatory framework in practice leads to the adoption of a narrow definition of health [12], based on few data, poorly specific and/or connected with health. An inconsistency between the intention stated within the general EIA/SEA approach and practice has been highlighted in the analysis in line with similar analyses by other authors [21]. While environmental sustainability pursues an integrated holistic approach in the assessment, in practice data availability is a fundamental limiting factor for a contextual and broad assessment of impact. Finally, a pattern of different implementation of deep and contextualized assessment is expected, depending on local (from regional to municipal level) availability of data, regarding both the environment and the health datasets. To encourage the use of assessment procedures based on a holistic health concept, more effort must be directed toward the definition of technical legislation and of adequate guidelines [49]. For this purpose, a synergistic contribution from all those working on the common ground of human health protection with different impact assessment tools is needed. 

The National Prevention Plan 2014-2018 acknowledges the shortcomings in the standards and instruments for the full application of the HIA. The plan calls for the development of tools for health assessments carried out by public health professionals to support the government in decisions related to the procedures for SEA and EIA.

Currently, a common definition of rules and tools within existing evaluation processes and hence toward homogeneity in health assessment is currently under development (“Tools for Health Impact Assessment—t4HIA” project, funded by the Ministry of Health in 2013), with the aim of identifying tools useful for developing the health component in EIA and SEA, for use by both public assessors and private proponents.
